# Transcaruncular Medial Wall Orbital Decompression: An Effective Approach for Patients with Unilateral Graves Ophthalmopathy

**DOI:** 10.1100/2012/312361

**Published:** 2012-04-30

**Authors:** Robert H. Hill, Craig N. Czyz, Thomas A. Bersani

**Affiliations:** ^1^Department of Ophthalmology, SUNY Upstate Medical University, 750 East Adams St., Syracuse, NY 13210, USA; ^2^Division of Ophthalmology, Section Oculofacial Plastic and Reconstructive Surgery, Ohio University/Doctor's Hospital, 5100 W. Broad St., Columbus, OH 43228, USA; ^3^Department of Ophthalmology, Oral and Maxillofacial Surgery, Grant Medical Center, 111 S. Grant Ave., Columbus, OH 43215, USA

## Abstract

*Purpose*. To evaluate the reduction in proptosis, incidence of postoperative diplopia, and postoperative globe symmetry after transcaruncular medial wall decompression in patients with unilateral Graves ophthalmopathy. *Methods*. Retrospective review of 16 consecutive patients who underwent unilateral transcaruncular medial wall orbital decompression from 1995 to 2007. The diagnosis of Graves ophthalmopathy was based on history and clinical findings including proptosis, lagophthalmos, lid retraction, motility restriction, and systemic thyroid dysfunction. *Results*. The mean reduction in proptosis was 2.3 mm. The mean difference in exophthalmometry preoperatively between the two eyes in each patient was 3.1 mm whereas postoperatively the mean difference was 1.1 mm (*P* = 0.0002). Eleven of 16 patients (69%) had 1 mm or less of asymmetry postoperatively. There was no statistically significant difference in the incidence of diplopia pre and postoperatively (*P* = 1.0). *Conclusions*. Medial wall orbital decompression is a safe and practical surgical approach for patients with unilateral Graves orbitopathy. The procedure carries a low risk of morbidity and yields anatomic retrusion of the globe that is comparable to other more invasive methods and may yield more symmetric postoperative results.

## 1. Introduction

Surgical decompression for Graves orbitopathy or Thyroid Eye Disease (TED) was originally reserved for those cases with unremitting optic neuropathy [1, 2]. However, orbital decompression has undergone an evolution, both in indication for surgery and also in methodology. Several surgical techniques including transcutaneous, transantral, endoscopic, and transcaruncular approaches to decompress the orbit, particularly the posterior aspect of the inferior and medial walls, have been developed [2–7]. Recent trends include fat decompression, more extensive posterior sculpting of the lateral wall, and various direct approaches to the medial wall.

 The associated social and psychological implications of proptosis have led to the use of orbital decompression for improved cosmesis [8]. This may be particularly true in patients with unilateral Graves ophthalmopathy. Some of the surgical techniques for orbital decompression can be more technically difficult and none are without the risk of postoperative diplopia. In our experience, we have found the transcaruncular medial wall decompression to provide excellent anatomical exposure, to be safe, and time efficient, it has a low incidence of postoperative complications, and it avoids an external scar. This study therefore evaluates the reduction in proptosis, incidence of postoperative diplopia, and postoperative globe symmetry after transcaruncular medial wall decompression in patients with unilateral Graves ophthalmopathy.

## 2. Methods

 From 1995 to 2007, 16 patients with unilateral Graves ophthalmopathy underwent transcaruncular medial wall orbital decompression. The medical records of these patients were retrospectively reviewed. The surgical indications for orbital decompression were cosmesis, exposure keratopathy, and limited ocular motility.

Demographics and preoperative characteristics were evaluated. History of smoking was noted, as this has been previously associated with greater incidence and increased severity of Graves disease [9–11]. Preoperative examination included Hertel exophthalmometry, examination of lid position, and presence of diplopia. The diagnosis of Graves ophthalmopathy was based on history and clinical findings including proptosis, lagophthalmos, lid retraction, motility restriction, and systemic thyroid dysfunction. Preoperative diplopia and most recent postoperative exophthalmometry readings were annotated. Preoperative and postoperative diplopia were considered “present” if the patient had diplopia in primary gaze.

Statistical analysis was performed with Prism 5 (GraphPad Software, Inc., La Jolla, CA,USA) statistical software. Outcome measures consisting of categorical data (diplopia) were analyzed using Fisher's exact test. A paired sample *t*-test was used to compare pre- and postoperative exophthalmometry values. Two-tailed testing was performed for all analysis and statistical significance for all tests was performed at the 0.05 alpha level.

### 2.1. Surgical Technique

**Figure 1:**
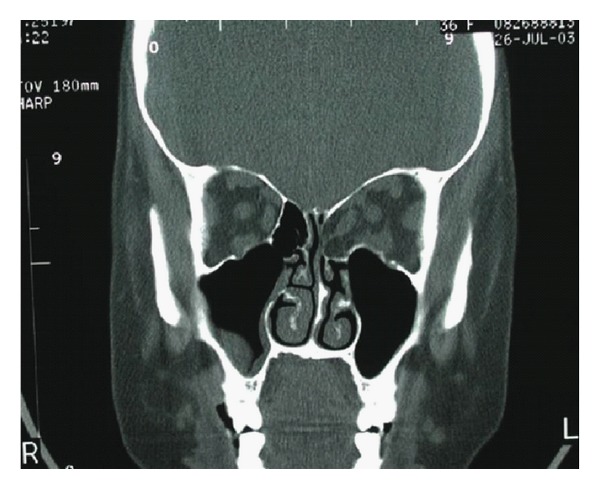
Coronal CT scan shows fat herniation into ethmoid sinus after transcaruncular medial wall orbital decompression.

Patients were placed under general endotracheal anesthesia after which local anesthetic was injected in the subconjunctival plane of the lower lid and medial canthus on the affected side. An incision was made vertically through the caruncle in the medial conjunctiva with dissection posteriorly through the subconjunctival tissue and then medially in the preseptal plane to the posterior lacrimal crest. The periosteum was then incised at the posterior lacrimal crest and the periorbita was reflected laterally exposing the medial orbital wall as far posteriorly as the orbital apex, as far superiorly as the frontal bone, and as far inferiorly as the mid orbital floor. The lamina papyracea was infractured and then the bone from the posterior lacrimal crest to the orbital apex and from the frontoethmoidal suture to the medial orbital floor was removed. The orbital floor directly inferior to the inferior rectus muscle was left intact to minimize the risk of postoperative diplopia. A total ethmoidectomy was performed. The periorbita was incised anteroposteriorly along the inferomedial orbit, and gentle globe retropulsion was performed to facilitate orbital fat herniation into the ethmoid sinus. The surgery was tailored to the degree of proptosis reduction desired. The bone was removed incrementally, periodically checking the degree of residual proptosis by viewing the patient from superiorly. The size of the periorbital incision was also tailored to control the amount of orbital fat herniation into the ethmoid sinus (Figure 1).

## 3. Results

 From 1995 to 2007, a total of 16 patients with unilateral Graves ophthalmopathy underwent transcaruncular medial wall orbital decompression. The mean age was 42.6 years (range 16 to 61 years); fourteen patients were female and two were male. Seven patients were smokers and nine patients were nonsmokers. Two patients received preoperative radiation therapy, while 14 patients did not receive radiation therapy (Table 1).

 The mean reduction in proptosis was 2.3 mm (SD = 0.5). The mean difference in exophthalmometry preoperatively between the two eyes in each patient was 3.1 mm (SD = 1.6) whereas postoperatively the mean difference was 1.1 mm (SD = 1.1). This difference was statistically significant (*P* = 0.0002). Eleven of 16 patients (69%) had 1 mm or less of asymmetry postoperatively (Table 2).

Of the seven patients with diplopia preoperatively, two (29%) had resolution of diplopia postoperatively. Two (22%) patients had new onset diplopia postoperatively. Neither of these patient required strabismus surgery for correction. There was no statistically significant difference in the incidence of diplopia pre- and postoperatively (*P* = 1.0).

## 4. Discussion

 There have been several other reports on the efficacy of transcaruncular orbital decompression in Graves disease; however none has established its efficacy in patients with unilateral disease [12–15]. We have found, in conjunction with others, that the transcaruncular approach to the medial orbit provides excellent anatomical exposure, is safe, time efficient, and has a low incidence of postoperative complications.

In our study, we found an average globe retrusion of 2.3 mm. Liao et al. reported a globe retrusion of 3.7 mm in their study of the transcaruncular approach for patients with Graves compressive optic neuropathy [14]. This increased retroplacement effect might be explained by the fact that the patients in their study were Asian. Typically Asians have small orbits which may lead to a higher percentage increase in orbital volume after decompression [16]. Also, we tailored the amount of surgery for symmetry and, in some cases, did less than the maximal amount possible.

**Figure 2:**
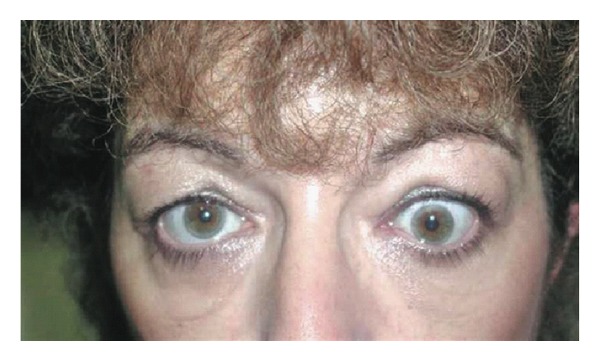
Preoperative photograph of patient with unilateral (left) Graves ophthalmopathy.

**Figure 3:**
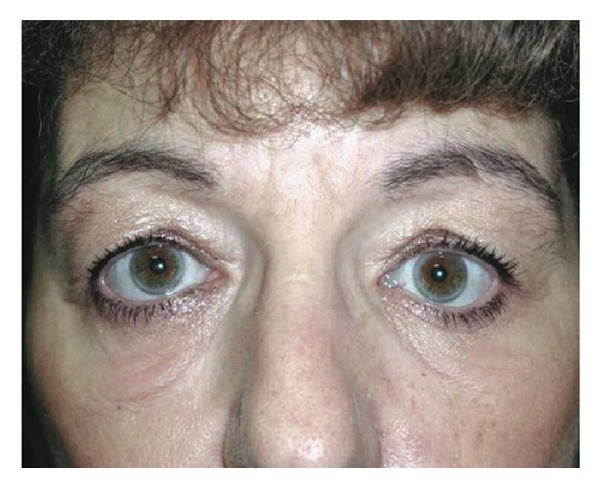
Postoperative photograph of same patient following decompression.

Other orbital decompression techniques report slightly higher retroplacement effects on the globe. Balanced orbital decompression by removal of the medial and lateral walls has been reported to give a retroplacement effect of 4.1 mm–5.3 mm [17, 18]. With 3-wall decompression, Ünal et al. found a mean reduction of proptosis of 6.9 mm [19]. Goldberg et al. report an average decompression of 4.5 mm with deep lateral wall decompression [20]. It is reasonable that transcaruncular orbital decompression may result in less retrusion of the globe than some other techniques. However, in this group of patients, the major goal was not maximal reduction in proptosis, but to achieve postoperative globe symmetry with a low incidence of complications. In this study 11 of 16 patients (69%) had 1 mm or less of asymmetry postoperatively, with no cases of visual loss (Figures 2 and 3).

 Of the seven patients with diplopia preoperatively, two (29%) had resolution of diplopia postoperatively; however two (22%) patients had new onset diplopia. Neither of these patients required strabismus surgery for correction. One reason that the transcaruncular medial wall approach may have a low incidence of new onset diplopia is that it allows for preservation of the anterior maxilloethmoidal strut, which decreases the risk of globe dystopia and new postoperative strabismus [21].

 In review of the literature, it seems that many now advocate the lateral wall as the primary wall for decompression. While this approach does have proven results, there is the potential to encounter the dura of the brain. Cerebral spinal fluid (CSF) leaks have been welldocumented during deep, posterior excavation of the lateral wall (greater wing of the sphenoid) during orbital decompression [18, 22, 23]. Dura can be encountered in three anatomical locations during lateral wall decompression: while drilling out the marrow space if the inner table of the sphenoid bone is penetrated, while drilling posterior to the marrow space where relatively thin bone overlies the middle cranial fossa, and inadvertently drilling superiorly through the orbital roof may expose dura in the anterior cranial fossa [24]. The potential for CSF leak can also be encountered with medial wall decompression through the cribriform plate [25]. This is particularly true for transantral and transnasal approaches but to our knowledge has not been reported in the transcaruncular approach.

In conclusion, transcaruncular medial wall orbital decompression is a safe and practical surgical approach for patients with Graves orbitopathy. Medial wall orbital decompression carries a low risk of morbidity including new onset motility disorders and yields anatomic retrusion of the globe that is comparable to other more invasive surgical methods. The results illustrate the procedure to be especially effective in achieving postoperative orbital symmetry in patients with unilateral Graves ophthalmopathy. In cases of Graves ophthalmopathy where exophthalmos exceeds 6 mm, additional procedures such as fat decompression or inclusion of other orbital walls should be considered for additional globe retrusion.

## Figures and Tables

**Table 1:** Patient demographics.

Patient	Age	Male/female	Smoker	Radiation
1	16	F	No	No
2	35	F	Yes	No
3	42	F	Yes	No
4	23	F	Yes	No
5	54	F	No	No
6	57	F	No	No
7	48	F	Yes	No
8	37	M	No	No
9	41	F	No	No
10	33	M	No	No
11	52	F	Yes	Yes
12	61	F	No	No
13	60	F	Yes	Yes
14	34	F	No	No
15	48	F	Yes	No
16	41	F	No	No

**Table 2:** Comparison of preoperative and postoperative exophthalmometry and diplopia in primary gaze.

Patient	Difference in exophthalmometry preop (mm)	Difference in exophthalmometry postop (mm)	Diplopia preop	Diplopia postop
1	2	1	No	No
2	2	1	No	No
3	2	0	Yes	No
4	4	0	Yes	No
5	1	1	No	Yes
6	5	0	No	No
7	2	1	No	No
8	4	2	No	No
9	2	0	No	Yes
10	2	0	No	No
11	5	1	No	No
12	3	2	Yes	Yes
13	6	2	Yes	Yes
14	4	1	Yes	Yes
15	5	4	No	No
16	1	2	Yes	Yes

	Mean = 3.13 mm	Mean = 1.13 mm		
